# Time‐of‐Day Effects on Postural Balance in Blind Children: A Cross‐Sectional Comparative Study

**DOI:** 10.1002/hsr2.72826

**Published:** 2026-07-14

**Authors:** Rym Baccouch, Rihab Borji, Rabeb Laatar, Achraf Ammar, Hamdi Chtourou, Haithem Rebai, Sonia Sahli

**Affiliations:** ^1^ Research Laboratory ‘Sports Performance Optimization, LR09SEP01 National Center of Medicine and Sport Sciences (CNMSS) Tunis Tunisia; ^2^ High Institute of Sport and Physical Education of Sfax University of Sfax Sfax Tunisia; ^3^ High Institute of Sport and Physical Education of Gafsa University of Gafsa Gafsa Tunisia; ^4^ Department of Training and Movement Science, Institute of Sport Science Johannes Gutenberg‐University Mainz Mainz Germany; ^5^ Research Laboratory, Molecular Bases of Human Pathology, LR19ES13, Faculty of Medicine of Sfax University of Sfax Sfax Tunisia; ^6^ Department of Nutrition and Food Technology, School of Agriculture The University of Jordan Amman Jordan; ^7^ Research Laboratory: Education, Motricité, Sport et Santé, EM2S, LR19JS01, High Institute of Sport and Physical Education of Sfax University of Sfax Sfax Tunisia; ^8^ Physical Activity, Sport, and Health National Observatory of Sport Tunis Tunisia

**Keywords:** attention, blind children, body temperature, muscular strength, postural balance, time‐of‐day

## Abstract

**Background and Aims:**

The effect of time‐of‐day on postural balance has been documented in sighted children. Given that vision is central to postural control and circadian synchronization, its absence may influence these temporal patterns. To date, the time‐of‐day effect on postural balance in blind children remains unexamined. Therefore, this study aimed to assess the effect of time‐of‐day on postural balance in blind children and to examine whether its temporal variation differs from the typical daily pattern observed in sighted peers.

**Methods:**

Two groups participated: 12 blind and 12 age‐matched sighted children. Postural balance was assessed using the center of pressure mean velocity (CoP_Vm_) during bipedal stance on firm and foam surfaces at 07:00, 12:00, 17:00, and 22:00 h. Quadriceps femoris muscle strength, auditory reaction time (ART), and oral temperature were also evaluated at each time point.

**Results:**

Blind children showed a significant nocturnal decrease in CoP_Vm_ at 22:00 h (*p* < 0.001), temporally aligned with a significant increase in lower limb strength (*p* < 0.001). Oral temperature was significantly lower at 07:00 h (*p* < 0.001), whereas no significant time‐of‐day effect was observed on ART (*p* > 0.99). In sighted children, at 17:00 h, CoP_Vm_ was significantly lower (*p* < 0.001), ART decreased significantly (*p* < 0.001), MVC increased significantly (*p* < 0.001), and oral temperature showed a significant peak (*p* < 0.001).

**Conclusion:**

Time‐of‐day influences postural balance in blind children, but with atypical fluctuations compared to sighted peers. Postural stability was lower during the diurnal phase in blind participants, highlighting the need for caution during balance‐demanding daily activities to reduce fall‐related injury risk.

## 1 Introduction

A biological rhythm is a cyclic variation in a specific function of a living organism [1]. Among these, circadian rhythms, lasting about 24 h, are the most studied in humans [2]. They are generated by the central clock in the suprachiasmatic nuclei (SCN) of the hypothalamus [3] and are synchronized to the 24‐h day by environmental cues, especially the light‐dark cycle [4, 5]. Light signals reach the SCN via the retinohypothalamic tract, regulating melatonin secretion by the pineal gland [6, 7, 8]. An intact visual system has been considered to be crucial for synchronizing the circadian system [9]. In blind individuals, the circadian rhythm is altered due to the absence of light [4, 10, 11]. As a result, their biological clock generates an endogenous cycle that is slightly longer than 24 h, leading to a condition known as free‐running [12]. Previous studies have reported a pattern of free‐running in hormone secretion and body temperature regulation in blind individuals [12, 13, 14]. Particularly, in blind adults, the secretion of testosterone, cortisol, melatonin, and growth hormone did not follow the usual light‐dark cycle as observed in their sighted counterparts [15]. Similarly, in totally blind adults, a free‐running rhythm has been observed in isometric contraction strength and reaction time [16]. Recently, blind children showed no diurnal variations in both maximal isometric hand contraction and resting oral temperature, further indicating a time‐of‐day pattern suggestive of circadian misalignment [17].

The visual system helps the human body assess external cues and adjust posture, and is therefore essential for postural control [18, 19]. Postural control is the ability to maintain or restore human balance by keeping the body's center of gravity over its base of support [20]. This function relies on the integration of visual, vestibular, and proprioceptive inputs by the central nervous system, with vision playing a most predominant role [18, 21, 22]. Vision informs the brain about body orientation [23] and its absence leads to impaired balance during both static and dynamic tasks [24, 25]. In addition, balance performance in individuals with visual impairments has been associated with physical activity level and anthropometric characteristics [26, 27]. In blind children, atypical motor development has been revealed resulting from the absence of sensory input through the visual pathway during infancy [28]. Notably, postural balance is often the most affected motor skill among blind children [29], increasing the risk of falls and limiting autonomy and social interaction [30, 31, 32].

The effect of time‐of‐day on postural control has been widely studied in normally sighted individuals across different age groups, showing that this motor skill varies throughout the day [33, 34, 35, 36, 37]. Postural performance peaks in the late afternoon, corresponding to circadian rhythms of body temperature [38] and vigilance [39, 40] in both young adults [36, 41] and children [33, 34]. In contrast, older adults showed lower postural performance at the late afternoon [37]. Postural control also depends on attentional resources [42] that peak in the late afternoon in sighted individuals [33, 34, 43, 44, 45]. Moreover, muscular strength, essential for maintaining balance [46], peaks in the afternoon according to synchronized circadian rhythm in sighted people, regardless of muscle type or body part involved [2, 47]. However, the time‐of‐day effect on postural balance in blind individuals is still unknown.

Given the important role of vision in circadian synchronization [9] and postural control [21], it is relevant to investigate whether postural balance varies across the day in blind children and whether this pattern differs from that of sighted peers. Moreover, postural balance is essential for daily activities [48], and understanding its daily variation may provide important clinical insights. Examining time‐of‐day effects on postural balance may help improve daily functioning and reduce fall‐related injury risk in blind children. If such temporal fluctuations in balance performance are confirmed, time‐of‐day should be taken into account as a confounding factor when evaluating postural control in this population. Furthermore, identifying periods of lowest balance performance could help adapted educators better schedule motor activities and balance training sessions. The current study aimed to explore the effects of time‐of‐day on postural balance in blind children compared to their sighted peers. It may be hypothesized that postural balance in blind children would display atypical temporal variations suggestive of circadian misalignment.

## 2 Materials and Methods

### 2.1 Participants

The G * power software (version 3.1.9.4; Kiel University, Kiel, Germany) [49] was used to calculate the required sample size. As there is a lack of published data concerning the time‐of‐day effects on postural balance in blinds, a medium effect size (Cohen *f* = 0.25), a statistical power of 0.80 and a significance level of 0.05 were assumed. To achieve the desired power, data from at least 24 participants was deemed to be sufficient.

Twelve blind participants and twelve age‐matched sighted children were recruited to participate in the current study Table 1. The recruitment process involved both advertising and contacting schools. The blind participants had complete congenital blindness, meaning they were unable to perceive any visual input in either eye [50]. Blind participants reported no impairments in other sensory modalities. Sighted participants had normal or corrected‐to‐normal vision. Participants were excluded if they had motor, neurological, attentional, or postural disorders, used medications, or had conditions affecting physical performance or circadian rhythms. All participants engaged only in recreational physical activity and had no prior sport training experience. This data was obtained through interviews with parents, teachers, and school personnel, as well as by reviewing educational and medical files. Prior to participation, all participants provided written consent from their parents or legal guardians to take part in the study and had the right to access their medical records. The current study was conducted in accordance with the Declaration of Helsinki, and approved by the Local Clinical Research Ethics Committee “Personal Protection Committee” under the following code (CPP SUD N° 0361/2021).

**Table 1:** Participant characteristics (mean ± standard deviation) of the blind group (BG) and control group (CG).

	BG (*n* = 12)	CG (*n* = 12)	*p* values
Height (cm)	127.8 ± 4.5	128.6 ± 4.3	0.91
Age (years)	7.4 ± 0.7	7.3 ± 0.8	1.00
Weight (kg)	28.7 ± 2.6	28.1 ± 3.9	0.74
Body mass index (kg/m^2^)	18.5 ± 3.3	18.1 ± 3.4	0.71

### 2.2 Experimental Design

To eliminate fear of the experimental equipment, ensure a clear understanding of the study procedures, and minimize any unexpected effects, all participants underwent familiarization sessions at different times of day during the 2 weeks preceding the experiment. During these sessions, participants' anthropometric data were also collected. Participants received detailed instructions regarding sleep and nutritional habits prior to the experiment to reduce potential confounding variables [51]. Guardians were specifically asked to maintain their children's usual sleep routines. In addition, masking factors such as the consumption of stimulant drinks (coffee), meals, and physical activity were carefully controlled [52]. Four test sessions were scheduled in randomized order: early morning (07:00 h), midday (12:00 h), late afternoon (17:00 h), and evening (22:00 h). For the morning session, participants were instructed to wake up at 06:00 h and were permitted to drink only one glass of water to minimize the effects of postprandial thermogenesis [51]. For the other sessions, participants were encouraged to eat breakfast at around 08:00 h, lunch at approximately 13:00 h, and dinner at around 18:00 h. A minimum interval of 4 h between the last meal and the test session was ensured. Water intake was permitted without any specific restrictions. During the 24 h preceding each experimental session, children were instructed to avoid any fatiguing activities that could influence the results. All assessments were conducted by professional experimenters with specialized expertise in evaluation procedures, who were not involved in data analysis to prevent experimenter bias. This study was conducted in spring 2022 (Mean temperature = 18°C ± 2.0°C).

The experimental protocol consisted of the evaluation of postural balance, lower limb muscle strength, auditory reaction time and oral temperature of the two groups throughout 4 times of the day. To minimize potential order, learning, and fatigue effects, the four experimental sessions (07:00, 12:00, 17:00, and 22:00 h) were administered using a randomized counterbalanced design. Four different session sequences were created, and participants from each group were randomly assigned to one of the sequences, with each sequence being performed by an equal number of participants. All sessions were separated by a recovery period of at least 36 h.

### 2.3 Measurements

#### 2.3.1 Postural Balance

Postural stability was evaluated using a stabilometric platform (PostureWin, Techno Concept, Cereste, France; 40 Hz frequency, 12‐bits A/D conversion), which consists of a steel plate mounted on three tri‐axial sensors that measure center of pressure (CoP) displacements. The stabilometric platform was calibrated according to the manufacturer's instructions prior to each testing session. During each trial, participants stood barefoot on the platform positioned 3 m from a wall, maintaining a relaxed upright stance with eyes open, arms at their sides, feet forming a 30° angle, and heels spaced 5 cm apart. The evaluations included two surface conditions: a rigid, non‐deformable platform and a foam surface using a block of foam (dimensions: 466 × 467 × 134 mm; density: 21.3 kg/m^3^; elasticity modulus: 20,900 N/m^2^) placed on the platform [53]. CoP sway data were thus collected under both firm and foam conditions. Each participant completed three trials per condition, with a 1‐min rest between attempts. The average of the three trials was used for statistical analysis. Each trial lasted 30 s. The CoP mean velocity (CoP_Vm_), calculated by dividing the total CoP displacement by the sampling duration, was chosen as postural balance parameter [54].

#### 2.3.2 Auditory Reaction Time

Auditory reaction time (ART) was measured using React software (version 1.0, WALLOPWARE) as previously described in children [55] and individuals with disability [56]. Participants were seated in front of a computer wearing earphones and a blindfold to ensure auditory stimulation only. They were instructed to press the spacebar with their dominant hand as quickly as possible after a beep sound. Ten trials were performed, and the mean ART was recorded in milliseconds (ms).

#### 2.3.3 Lower Limb Muscular Strength

The isometric strength of the quadriceps femoris muscle was evaluated using a fixed dynamometry device considered as a safe and simple method of assessing muscle strength, previously used in children [57]. In this study, a connected electronic traction dynamometer (K‐Force Link, KINVENT, Montpellier, France) was employed, enabling quick and objective data collection. The device was linked via Bluetooth to the “K FORCE” application (version 5.4.10), pre‐installed on a personal smartphone, allowing for real‐time recording of strength measurements. The dynamometer calibration and Bluetooth synchronization were verified before each assessment session according to the manufacturer's guidelines. Participants were assessed in a standardized seated position, with arms crossed and with the hip and knee angles fixed at 90° from full extension (= 0°). A cuff was placed ~2 cm above the lateral malleolus and fixed to a dynamometer secured to the table. After a brief warm‐up, participants performed 3 maximum voluntary contractions (MVC) of the quadriceps, each lasting 5 s, with 2 min rest. Standardized verbal encouragement was given. The dominant limb was tested, and the highest value was retained as the representative MVC, in Newtons of force (N).

#### 2.3.4 Body Temperature

Oral temperature was assessed with a digital clinical thermometer (Omron, Paris, France; accuracy ±0.05°C). The thermometer was placed sublingually for at least 3 min at the beginning of each testing session, following a seated rest period of at least 15 min.

### 2.4 Statistical Analysis

Statistical analyses were performed using STATISTICA for Windows software (version 8.0; StatSoft Inc., Tulsa, OK). Results were expressed as means (m) ± standard deviations (SD). The Shapiro–Wilk test was used to assess the normality of the data and the Levene's test was employed to evaluate the homogeneity of variance. After confirming the normal distribution and the homogeneity of variance, parametric tests were utilized. Group comparisons for age and anthropometric variables were conducted using independent sample *T*‐tests.

The CoP_Vm_ was analyzed using a three‐way analysis of variance (ANOVA) with repeated‐measures (2 group × 4 time × 2 surface). The group factor had 2 levels: Blind group (BG) and control group (CG). The time factor had 4 levels: 07:00 h, 12:00 h, 17:00 h and 22:00 h. The surface factor had 2 levels: firm and foam. The group is as a between factor; the time and surface are as within factors. To analyze oral temperature, ART and MVC values, a two‐way ANOVA with repeated measures (2 group × 4 time) was applied. For each statistically significant effect of the main factor and interaction, a post hoc analysis was performed using the Bonferroni test. For all statistical analyses, the alpha level of statistical significance was set as *p* < 0.05. Effect sizes were calculated as partial eta squared (*η_p_* ^2^) to assess the practical significance of our findings (small effect: 0.01 < *η_p_* ^2^ < 0.06; medium effect: 0.06 < *η_p_* ^2^ < 0.14; large effect: *η_p_* ^2^ > 0.14) [58, 59]. In addition, the mean differences (MD) and the 95% confidence intervals (CI) were also calculated [60].

## 3 Results

### 3.1 Postural Balance

The three‐way ANOVA with repeated measures showed a significant group (*F* = 234.90, *p* < 0.001, *η*_*p*_^*2*^ = 0.91), time (*F* = 23.06, *p* < 0.001, *η*_*p*_^*2*^ = 0.51) and surface (*F* = 113.88, *p* < 0.001, *η*_*p*_^*2*^ = 0.84) main effects on the CoP_Vm_. Moreover, significant time x group (*F* = 21.69, *p* < 0.001, *η*_*p*_^*2*^ = 0.50) and surface x group (*F* = 18.31, *p* < 0.001, *η*_*p*_^*2*^ = 0.45) interactions on the CoP_Vm_ were observed.

For the BG, in both surfaces, the post hoc test showed that the CoP_Vm_ decreased significantly at 22:00 h compared to all the other times of testing (*p* < 0.001; 22:00 h vs. 07:00 h; MD = 3.52 [95% CI: 2.26, 4.79], 22:00 h vs. 12:00 h; MD = 3.36 [95% CI: 2.09, 4.62], 22:00 h vs. 17:00 h; MD = 2.52 [95% CI: 1.25, 3.78]). No significant variation in the CoP_Vm_ was observed between 07:00 h and 12:00 h (*p* > 0.99; MD = 0.16 [95% CI: −1.10, 1.42]), or between 12:00 h and 17:00 h (*p* = 0.94; MD = 0.84 [95% CI: −0.42, 2.10]). For the CG, in both surfaces, the Bonferroni test demonstrated that the CoP_Vm_ was significantly lower at 17:00 h compared to all the other times of testing (*p* < 0.001; 17:00 h vs 07:00 h; MD = 1.69 [95% CI: 0.43, 2.96], 17:00 h vs. 12:00 h; MD = 1.80 [95% CI: 0.54, 3.07], 17:00 h vs. 22:00 h; MD = −1.55 [95% CI: −2.82, −0.29]).

**Figure 1:**
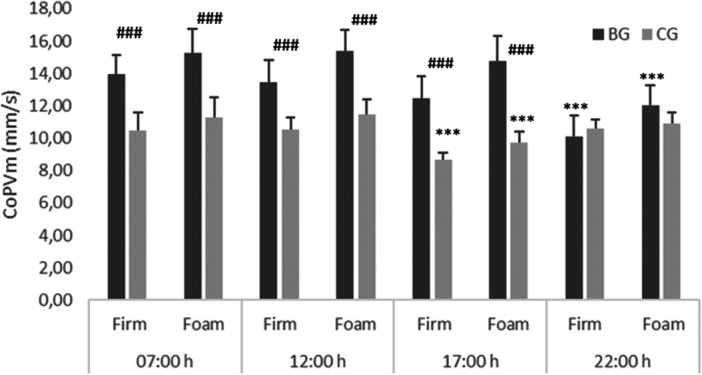
Center of pressure mean velocity (CoP_Vm_) on Firm and Foam surfaces, for the blind group (BG) and the control group (CG) recorded at 07:00, 12:00, 17:00 and 22:00 h. ***significant differences between test sessions at *p* < 0.001; ^###^significant differences between BG and CG at *p* < 0.001.

Moreover, in both surfaces, the post hoc test revealed significant higher CoP_Vm_ values in the BG compared to the CG at 07:00 h, 12:00 h and 17:00 h (*p* < 0.001; at 07:00 h; MD = 3.72 [95% CI: 2.44, 5.01], at 12:00 h; MD = 3.45 [95% CI: 2.17, 4.73], at 17:00 h; MD = 4.42 [95% CI: 3.13, 5.70]). This difference between the two groups was no significant (*p* > 0.99; MD = 0.34 [95% CI: −0.94, 1.62]) at 22:00 h Figure 1.

### 3.2 Auditory Reaction Time

The two‐way ANOVA with repeated measures revealed a significant time (*F* = 4.07, *p* = 0.01, *η*_*p*_^*2*^ = 0.16) main effect as well as a significant time x group (*F* = 6.30, *p* < 0.001, *η*_*p*_^*2*^ = 0.22) interaction on the ART.

For the BG, no significant variation in ART was observed across the different testing times (*p* > 0.99; 22:00 h vs. 07:00 h; MD = −6.21 [95% CI: −48.72, 36.31], 22:00 h vs. 12:00 h; MD = 1.50 [95% CI: −41.01, 44.01], 22:00 h vs. 17:00 h; MD = 5.42 [95% CI: −37.09, 47.94]). For the CG, the Bonferroni test showed that the ART was significantly decreased at 17:00 h compared to all the other times of testing (*p* = 0.002; 17:00 h vs. 07:00 h; MD = 54.50 [95% CI: 11.98, 97.01], 17:00 h vs. 12:00 h; MD = 55.33 [95% CI: 12.81, 97.85], *p* < 0.001; 17:00 h vs. 22:00 h; MD = −64.25 [95% CI: −106.76, −21.73]) Table 2. Moreover, the post hoc test revealed no significant difference between the two groups in terms of ART values across the different testing times (*p* = 0.79; at 07:00 h; MD = −28.62 [95% CI: −71.25, 14.00], *p* > 0.99; at 12:00 h; MD = −21.75 [95% CI: −64.37, 20.87], *p* = 0.12; at 17:00 h; MD = 37.51 [95% CI: −5.11, 80.13], *p* = 0.39; at 22:00 h; MD = −32.16 [95% CI: −74.79, 10.46]).

**Table 2:** Mean values (±SD) of auditory reaction time (ART), maximum voluntary contraction (MVC) and oral temperature for the blind group (BG) and the control group (CG) recorded at 07:00, 12:00, 17:00 and 22:00 h.

		Testing sessions			
		07: 00 h	12: 00 h	17: 00 h	22: 00 h
ART (ms)	CG	276.50 ± 30.30	277.33 ± 21.59	222.00 ± 15.86***	286.25 ± 39.20
	BG	247.87 ± 32.42	255.58 ± 36.05	259.51 ± 35.31	254.08 ± 33.63
MVC (N)	CG	124.46 ± 10.07^###^	126.04 ± 7.76^###^	138.60 ± 9.29***^###^	130.16 ± 9.53^###^
	BG	94.86 ± 9.74	98.27 ± 7.23	100.13 ± 7.26	106.60 ± 7.67***
Oral temperature (°C)	CG	36.19 ± 0.18***	36.75 ± 0.23***	37.18 ± 0.16***#	36.65 ± 0.22***^###^
	BG	36.30 ± 0.21***	36.87 ± 0.17	36.86 ± 0.22	37.09 ± 0.27

*Note:* ***Significant differences between test sessions at *p* < 0.001; ^#, ###^significant differences between BG and CG at *p* < 0.05 and *p* < 0.001, respectively.

### 3.3 Lower Limb Muscular Strength

The two‐way ANOVA with repeated measures demonstrated significant group (*F* = 86.90, *p* < 0.001, *η*_*p*_^*2*^ = 0.80) and time (*F* = 30.38, *p* < 0.001, *η*_*p*_^*2*^ = 0.58) main effects and a significant time x group (*F* = 13.39, *p* < 0.001, *η*_*p*_^*2*^ = 0.38) interaction on the MVC.

For the BG, the post hoc test showed that the MVC increased significantly at 22:00 h compared to all the other times of testing (*p* < 0.001; 22:00 h vs. 07:00 h; MD = −11.74 [95% CI: −17.32, −6.15], 22:00 h vs. 12:00 h; MD = −8.32 [95% CI: −13.91, −2.74], *p* = 0.01; 22:00 h vs. 17:00 h; MD = −6.46 [95% CI: −12.05, −0.88]). No significant variation in MVC values during the diurnal phase (*p* > 0.99; 07:00 h vs. 12:00 h; MD = −3.41 [95% CI: −9.00, 2.17], 12:00 h vs. 17:00 h; MD = −1.86 [95% CI: −7.44, 3.72]) was observed. For the CG, the Bonferroni test revealed that the MVC was significantly increased at 17:00 h compared to all the other times of testing (*p* < 0.001; 17:00 h vs. 07:00 h; MD = −14.14 [95% CI: −19.72, −8.55], 17:00 h vs. 12:00 h; MD = −12.56 [95% CI: −18.15, −6.98], 17:00 h vs. 22:00 h; MD = 8.44 [95% CI: 2.85, 14.02]). Furthermore, the post hoc test revealed significant lower MVC values in the BG compared to the CG at all the times of testing (*p* < 0.001; at 07:00 h; MD = −29.60 [95% CI: −41.95, −17.24], at 12:00 h; MD = −27.76 [95% CI: −40.11, −15.41], at 17:00 h; MD = −38.47 [95% CI: −50.82, −26.11], at 22:00 h; MD = –23.56 [95% CI: −35.91, −11.20]) Table 2.

### 3.4 Body Temperature

The two‐way ANOVA with repeated measures showed a significant time (*F* = 71.00, *p* < 0.001, *η*_*p*_^*2*^ = 0.76) main effect and a significant time x group (*F* = 15.00, *p* < 0.001, *η*_*p*_^*2*^ = 0.40) interaction on the oral temperature.

For the BG, the post hoc test showed that the oral temperature was significantly lower at 07:00 h compared to all the other times of testing (*p* < 0.001; 07:00 h vs. 12:00 h; MD = −0.57 [95% CI: −0.84, −0.31], 07:00 h vs 17:00 h; MD = −0.56 [95% CI: −0.83, −0.30], 07:00 h vs. 22:00 h; MD = −0.79 [95% CI: −1.05, −0.52]). However, no significant differences between 12:00 h and 17:00 h (*p* > 0.99; MD = 0.008 [95% CI: −0.25, 0.27]); and between 17:00 h and 22:00 h (*p* = 0.19; MD = −0.22 [95% CI: −0.48, 0.03]) were observed in terms of oral temperature. For the CG, the Bonferroni test revealed that the oral temperature was significantly lower at 07:00 h compared to all the other times of testing (*p* < 0.001; 07:00 h vs. 12:00 h; MD = −0.55 [95% CI: −0.82, −0.29], 07:00 h vs. 17:00 h; MD = −0.99 [95% CI: −1.25, −0.72], 07:00 h vs. 22:00 h; MD = −0.45 [95% CI: −0.72, −0.19]). The oral temperature increased significantly at 17:00 h compared to all the other times of testing (*p* < 0.001; 17:00 h vs. 07:00 h; MD = −0.99 [95% CI: −1.25, −0.72], 17:00 h vs. 12:00 h; MD = −0.43 [95% CI: −0.69, −0.17], 17:00 h vs. 22:00 h; MD = 0.53 [95% CI: 0.27, 0.79]). In addition, the post hoc test demonstrated significant (*p* = 0.02; MD = −0.31 [95% CI: −0.61, −0.02]) higher oral temperature at 17:00 h in the CG compared to the BG. In contrast, at 22:00 h, the oral temperature was significantly (*p* < 0.001; MD = 0.44 [95% CI: 0.14, 0.74]) higher in the BG compared to the CG. The difference between the two groups was no significant at 07:00 h (*p* > 0.99; MD = 0.11 [95% CI: −0.19, 0.40]) and at 12:00 h (*p* > 0.99; MD = 0.12 [95% CI: −0.17, 0.42]) in terms of oral temperature (Table 2).

## 4 Discussion

This study investigated how different times of day influence postural balance, muscular strength, ART and body temperature in blind children compared to their sighted counterparts.

Blind children showed no diurnal variation in postural balance, but a significant improvement was observed during the evening session. These results suggested that blind children may exhibit a time‐of‐day pattern suggestive of circadian misalignment. In fact, in blind children, the postural performance peak occurred in the evening rather than in the late afternoon, as found in sighted peers. The observed time‐of‐day variations in postural balance, in sighted children, are consistent with previous studies conducted in children and young adults [33, 34, 36, 41]. However, to date, studies examining the influence of time‐of‐day on postural balance in blind individuals remain rare.

This atypical variation in postural balance observed in blind children may be partly explained by fluctuations in muscle force, which plays a key role in maintaining postural stability [61], as it coincided with similar variations in lower limb muscular strength. In sighted children, the observed peak in postural performance at 17:00 h coincided with the highest levels of quadriceps muscular strength and oral temperature recorded at the same time. Similarly, a previous study showed that muscle strength, particularly in the lower limbs, peaks in the late afternoon in sighted individuals [47]. Importantly, in blind children, the ART was stable across all testing times, showing no significant time‐of‐day effects. Supporting our observations, previous research in blind young adults has reported desynchronized circadian rhythms of simple reaction time, characterized by a free‐running pattern, longer than 24 h [16]. However, the absence of time‐of‐day effects in ART in blind children should be interpreted with caution. The use of a simple reaction time task may have limited sensitivity to detect subtle circadian fluctuations, as such paradigms are susceptible to ceiling effects and may not adequately reflect higher‐order attentional processes. In sighted children, the best reaction time values were observed at 17:00 h, coinciding with peak postural performance. This finding is consistent with previous studies suggesting that attentional capacity is time‐of‐day dependent and tends to improve in the middle morning and late afternoon in sighted children [33, 34] and young adults [43, 55]. Considering that postural control relies on attentional resources [42] it may be suggested that the best attentional capacity observed at 17:00 h could explain the enhanced postural performance in sighted children. Furthermore, in the present study, sighted children have showed a synchronized oral temperature rhythm that align with earlier studies conducted in both sighted children [33, 34, 62, 63] and adults [64, 65], reporting that oral temperature values were significantly higher in the evening compared to the morning [64]. In contrast, blind children in this study exhibited an unusual time‐of‐day pattern of oral temperature. Previously, in blind adults, body temperature rhythms have been shown to follow atypical time‐of‐day pattern, with a progressive daily delay in the timing of the temperature peak [16]. In addition, a prior investigation in visually impaired and totally blind children reported no significant differences in oral temperature between morning and late afternoon test sessions [17].

Although the present study did not directly investigate the underlying mechanisms responsible for the observed time‐of‐day fluctuations in postural balance among blind children and did not assess hormonal or circadian biomarkers, several physiological processes may speculatively explain these findings. In blind individuals, previous literature suggests that circadian rhythm disturbances may result from impaired light perception and genetic influences affecting phase regulation [66, 67]. The light–dark cycle is considered a key synchronizer of the human biological clock [9]. In the absence of light perception, melatonin regulation may be disrupted, leading to circadian misalignment and altered sleep–wake cycles [4, 68]. Free‐running sleep–wake rhythms and delayed phase patterns have been reported in blind individuals [69, 70]. These circadian alterations may contribute to atypical physical and postural performance in blind populations [15], as well as to impaired daily functioning, increased daytime sleepiness [71, 72], and reduced alertness [11, 73]. Furthermore, Melatonin has been suggested to influence thermoregulation by promoting heat loss and lowering core body temperature in the evening [74]. Therefore, impaired or desynchronized melatonin secretion in blind individuals may contribute to altered temperature regulation [73]. Previous findings in blind adults have also suggested an association between melatonin rhythm and motor performance [15]. In sighted individuals, melatonin follows a well‐defined nocturnal secretion pattern [75]. Although melatonin was not measured in the present study, its dysregulation may partly explain the atypical evening improvement in postural balance observed in blind children.

In the present study, the atypical oral temperature pattern observed in blind children may potentially reflect altered time‐of‐day variation that could be suggestive of circadian misalignment. Given the close relationship between body temperature and circadian regulation [38], this altered pattern might also be associated with the atypical fluctuations in postural balance observed in blind children. In the other hand, in sighted individuals, previous studies have suggested that circadian increases in body temperature may enhance muscle performance through improved conduction velocity and reduced intramuscular resistance [58, 65, 76]. However, passive warming studies indicated that temperature alone cannot fully explain late‐afternoon strength peaks [77]. It has been suggested, therefore, that body temperature and muscular strength rhythms may be co‐regulated by the central circadian clock (SCN) without a direct causal link [62, 77, 78].

When comparing the two groups, blind children demonstrated lower postural balance performance than their sighted peers during the diurnal phase. This deficit may be attributed to atypical motor development resulting from the absence of visual cues during early infancy [28] and the lack of consistent visual inputs indispensable for spatial orientation and postural control [29, 79]. However, this difference between the two groups was not significant during the nocturnal test session (22:00 h), which may be explained by the observed peak in postural balance performance among blind children at that time, coinciding with a decline in performance among sighted children. Regarding muscular strength, blind children consistently demonstrated lower values compared to sighted peers across all testing times, despite an observed peak in their muscular strength at 22:00 h and a concurrent decline in the sighted group. In this context, previous studies have shown that individuals with visual impairments or blindness often display muscle weakness [80] which is attributed not only to atypical motor development [28], but also to reduced opportunities for engaging in physical activity and sports [81]. Longitudinal evidence in Paralympic athletes has also highlighted changes in motor performance and physiological characteristics over time [82], reinforcing the role of physical training and activity in individuals with vision loss [83]. Therefore, the distinct time‐of‐day effects on postural balance observed between blind and sighted children may reflect deficits in both postural control and muscular strength among the blind group. In contrast, current findings showed that the difference between the two groups in terms of attentional capacity was no significant at all the times of testing.

### 4.1 Limitations and Future Studies

Although this study provides important insights, several limitations should be acknowledged. First, postural balance was assessed only under static conditions; future studies should include dynamic balance and gait assessments that may better reflect daily activities. Second, the study examined time‐of‐day effects on behavioral and physiological variables without directly assessing endogenous circadian rhythms. Although potential mechanisms involving sleep–wake rhythms, melatonin, and cortisol were discussed, these variables were not measured and the proposed explanations therefore remain speculative. In addition, potential confounders such as adherence to pre‐test instructions, sleep quality, and fatigue were not objectively monitored. Future studies should incorporate objective assessments of sleep–wake patterns, fatigue, and biological circadian markers to better clarify the mechanisms underlying the atypical time‐of‐day patterns observed in blind children. Third, although oral temperature is a practical and non‐invasive measure in pediatric populations, it remains an indirect marker of circadian rhythms and may be influenced by external factors. Future research should therefore include gold‐standard circadian markers such as core body temperature or melatonin. Fourth, the use of a simple auditory reaction time task may have limited sensitivity to detect subtle circadian variations in attentional performance. More complex cognitive assessments, such as choice reaction time or dual‐task paradigms, should be considered in future studies. Fifth, blindness classification was based on school medical records without detailed ophthalmological documentation, preventing assessment of residual light perception and etiological subgroup analyses. Future studies should therefore include detailed ophthalmological characterization to better interpret circadian and postural responses in individuals with visual impairments. Finally, although large effect sizes were observed, the relatively small sample size may increase the risk of effect overestimation and limit generalizability. In addition, the sample included only recreationally active children with congenital total blindness and without comorbidities; therefore, the findings may not extend to children with acquired blindness, partial visual impairment, or additional disorders. Future studies, with larger samples should examine more diverse clinical populations.

## 5 Conclusion

The present study demonstrated an atypical time‐of‐day pattern in postural balance in blind children, characterized by stable daytime values and improved evening performance compared with sighted peers. In blind children, postural balance variations appeared to coincide with changes in lower leg muscular strength, whereas attentional capacity remained stable and oral temperature showed an atypical pattern. These findings highlight the importance of considering time‐of‐day when assessing postural balance and designing rehabilitation programs for blind children. In addition, impaired daytime balance may increase fall‐related injury risk during daily activities. Simple assessments such as postural performance, oral temperature, reaction time, and muscular strength may help educators and clinicians identify atypical time‐of‐day patterns and guide appropriate management strategies.

## Author Contributions

**Rym Baccouch:** conceptualization, data curation, formal analysis, investigation, visualization, writing – original draft, methodology. **Rihab Borji:** investigation, methodology, formal analysis, writing – review and editing. **Rabeb Laatar:** conceptualization, methodology, data curation, project administration, writing – review and editing. **Achraf Ammar:** validation, visualization, project administration, writing – review and editing. **Hamdi Chtourou:** validation, supervision, writing – review and editing. **Haithem Rebai:** supervision, writing – original draft, project administration. **Sonia Sahli:** methodology, supervision, project administration, writing – review and editing.

## Funding

The authors have nothing to report.

## Ethics Statement

Prior to participation, all participants provided written consent from their parents or legal guardians to take part in the study. The current study was conducted in accordance with the Declaration of Helsinki, and approved by the Local Clinical Research Ethics Committee ‘Personal Protection Committee’ under the following code (CPP SUD N° 0361/2021).

## Conflicts of Interest

The authors declare no conflicts of interest.

## Transparency Statement

Rym Baccouch affirms that this manuscript is an honest, accurate, and transparent account of the study being reported; that no important aspects of the study have been omitted; and that any discrepancies from the study as planned (and, if relevant, registered) have been explained.

## Data Availability

The data that support the findings of this study are available from the corresponding author upon reasonable request.
